# Non-adherence to drug therapy and drug acquisition costs in a national population - a patient-based register study

**DOI:** 10.1186/1472-6963-11-326

**Published:** 2011-11-28

**Authors:** Bo Hovstadius, Göran Petersson

**Affiliations:** 1 eHealth Institute, Linnaeus University, Kalmar, Sweden; 2 School of Natural Sciences, Linnaeus University, Kalmar, Sweden; 3 School of Health and Caring Sciences, Linnaeus University, Kalmar, Sweden

## Abstract

**Background:**

Patients' non-adherence to drug therapy is a major problem for society as it is associated with reduced health outcomes. Generally, approximately only 50% of patients with chronic disease in developed countries adhere to prescribed therapy, and the most common non-adherence refers to chronic under-use, i.e. patients use less medication than prescribed or prematurely stop the therapy. Patients' non-adherence leads to high additional costs for society in terms of poor health. Non-adherence is also related to the unnecessary sale of drugs. The aim of the present study was to estimate the drug acquisition cost related to non-adherence to drug therapy in a national population.

**Methods:**

We constructed a model of the drug acquisition cost related to non-adherence to drug therapy based on patient register data of dispensed out-patient prescriptions in the entire Swedish population during a 12-month period. In the model, the total drug acquisition cost was successively adjusted for the assumed different rates of primary non-adherence (prescriptions not being filled by the patient), and secondary non-adherence (medication not being taken as prescribed) according to the patient's age, therapies, and the number of dispensed drugs per patient.

**Results:**

With an assumption of a general primary non-adherence rate of 3%, and a general secondary non-adherence rate of 50%, for all types of drugs, the acquisition cost related to non-adherence totalled SEK 11.2 billion (€ 1.2 billion), or 48.5% of total drug acquisition costs in Sweden 2006.

With the assumption of varying primary non-adherence rates for different age groups and different secondary non-adherence rates for varying types of drug therapies, the acquisition cost related to non-adherence totalled SEK 9.3 billion (€ 1.0 billion), or 40.2% of the total drug acquisition costs.

When the assumption of varying primary and secondary non-adherence rates for a different number of dispensed drugs per patient was added to the model, the acquisition cost related to non-adherence totalled SEK 9.9 billion (€ 1.1 billion), or 42.6% of the total drug acquisition costs.

**Conclusions:**

Our estimate indicates that drug acquisition costs related to non-adherence represent a substantial proportion of the economic resources in the health care sector. A low rate of primary non-adherence, combined with a high rate of secondary non-adherence, contributes to a large degree of unnecessary medical spending. Thus, efforts of different types of interventions are needed to improve secondary adherence.

## Background

Patients' non-adherence to drug therapy is a major problem in health care as this is associated with reduced health outcomes [1-3]. Patients' adherence to prescribers' drug therapy is essential for treatment efficacy, patient safety, and healthcare costs. If patients do not follow the prescribed drug therapy the drugs will have no, or a reduced, effect. A large proportion of patients in all ages with different diseases do not adhere to therapy instructions. In developed countries, non-adherence to long-term therapy for chronic diseases averages 50% [4-6], and in developing countries the non-adherence rate is even higher [5].

There are several determinants as regards patients' non-adherence [4]: complexity of therapy [5], duration of therapy [5], characteristics of disease [5,7], adverse drug reactions [5,8], cost of treatment [5,7,9], characteristics of health service provision[5], interaction between prescriber and patient [5,10], prescribers follow-up [5,11], multiple providers [5,10] socio-economic variables [5], multiple medication [10], the patients' own view of required therapy [8,12,13] and unintended non-adherence [12]. The most common non-adherence refers to chronic under-use, i.e. patients use less medication than prescribed, or prematurely stop the therapy [5]. The World Health Organization (WHO) categorised the determinants of non-adherence into five dimensions: social and economic, health system-related, therapy-related, condition-related, and patient-related [5].

Non-adherence is commonly divided into primary and secondary non-adherence. Irrespective of the cause, primary non-adherence represents the prescriptions the patient not fill at pharmacies [14,15] and secondary non-adherence represents the dispensed drugs that the patient not take as prescribed [15,16].

The rate of non-adherence differs between different age groups. Generally, the rate of non-adherence decreases with a higher age [7,8,11,12,17]. There is a minor gender difference in non-adherence; the rate of non-adherence is slightly higher for women than for men [8,18,19].

Non-adherence is a problem across all therapeutic areas [5,20,21]. However, variation in non-adherence can, to some extent, be explained by the type of drug or in terms of whether the patient's drug therapy is chronic or acute [22-25].

The use of multiple medication is a rational therapy for many patients, but it is also a well-known risk factor for patients' health as multiple medication is associated with adverse drug reactions, interactions and non-adherence to drug therapy [26], and the rate of non-adherence increases with an increasing number of drugs [27-35].

Cost-related non-adherence (CRNA) is a major determinant of non-adherence, [18,36]. Countries with the lowest out-of pocket costs for drugs also have the lowest average rate of CRNA [36]. Countries with no mandatory health insurance system also have large internal variations in CRNA [9,17].

Non-adherence leads to poor health outcomes and high extra costs for society when filled drug prescriptions cannot produce an effect as they are not taken by the patient [37]. Non-adherence also represents a waste of substantial economic resources due to unnecessary drug sales [16]. In the majority of countries, the cost of wasted medication is shared between the patients and society.

With access to the Swedish prescribed drug register, it is possible to utilise patient-based data for an entire national population in order to analyse drug acquisition costs in terms of drug groups, age, gender, and multiple medication, without any sample, recall, or interview bias. With patient-based data on dispensed drugs, it is possible to shift the focus from the drug exposure of the total population, e.g. acquisition cost per inhabitant, to the drug exposure of the patients who have actually received different medications, e.g. acquisition cost per patient on the basis of a certain drug.

### Aim of the study

To estimate the drug acquisition costs related to non-adherence to drug therapy in a national population.

## Methods

To estimate the drug acquisition costs related to non-adherence to drug therapy in a national population, we studied the patient based data of all dispensed prescription drugs in the entire Swedish population during a 12-month period 2006. This data was extracted from the Swedish prescribed drug register [38].

### The Swedish prescribed drug register

The Swedish prescribed drug register is patient based and contains data for dispensed out-patient prescriptions at all Swedish pharmacies from July 1, 2005. The register is complete with regard to dispensed prescriptions. In 2006, dispensed out-patient prescriptions accounted for 82% of all defined daily doses (DDD), and the remaining 18% consisted of OTC sales and inpatient use in hospitals [38].

The registration is mandatory and the following data from the register was used in our study: number of patients, the dispensed drug (substance), number of dispensed prescription drugs per patient, the cost of the drug, DDD, number of DDD per patients, the date of dispensing, age, gender, and a unique identifier (personal identification number) of the patient.

The study population was comprised of all patients of all ages receiving one or more dispensed drugs during the 12-month study period that is, a total of, 6, 161, 673 (3, 481, 371 women and 2, 680, 302 men), corresponding to 67.6% of the Swedish population [39].

The applied definition of drug was the chemical entity or substance comprising the fifth level in the WHO Anatomic, Therapeutic, Chemical (ATC) classification 2006. In the presentation, the drugs were categorized according to the second level of the ATC-classification.

All data processing was undertaken anonymously without the personal identification number. Only gender and age, originally embedded in the personal identification number, were recorded. The study population was stratified by gender and age (10-year classes). The results were compared to the number of individuals per gender and age group in the Swedish population.

In Sweden, prescriptions are valid for 12 months and the filling of one prescription usually covers consumption for a 3-month period [40].

### Adherence

Adherence can be defined as "the extent to which the patient follows medical instructions" [5], or "the extent to which patient behaviour corresponds with recommendations from a health care provider" [5].

### Primary and secondary adherence

Medical adherence is commonly divided into primary adherence (prescription being filled by patient) [14,15,41] and secondary adherence (medication used as prescribed) [15,16,41].

Adherence measurement options include drug claims data, interviews, surveys, pill counts and drug assays. The majority of adherence research has addressed secondary adherence of chronic therapies [42]. There exist a large number of varying measurements to estimate secondary adherence of long-term therapies from administrative data, including e.g. MRA, MPR, and PDC [43].

#### A model to estimate the share of drug costs related to non-adherence

In order to estimate the share of drug costs related to non-adherence, we constructed a model based on the patient data of all dispensed prescription drugs in the entire Swedish population during a 12-month period, 2006. In the model, the total drug acquisition cost was successively adjusted for assumptions regarding different rates of primary non-adherence and secondary adherence, based on the patient's age, therapies, and the number of dispensed drugs per patient. As secondary non-adherence refers to the amount of prescribed drugs and not to the patients' filled prescriptions, the drug cost related to non-adherence is defined as the difference in cost between actual, filled prescriptions (primary adherence) and a calculated estimation of the total cost of the amount of drugs taken as prescribed (secondary adherence).

### Assumptions of non-adherence

Based on two previous studies from Sweden, we applied a primary non-adherence rate of 3% (measured as non-filled prescriptions at pharmacies) in the model [12,44]. The applied rate of primary non-adherence is also equivalent to the proportion of the population which did not fill a prescription during 2005 [18].

### Gender

Generally, there is a slight gender difference in adherence [8,19], but studies from Sweden have reported contradictory results [12,18]. Thus, no gender adjustment was implemented in our model.

### Age

Based on previous studies [7,11,12,17,20,45], we also adjusted our model for age difference in primary adherence. The highest rate of adherence is displayed for the elderly and children. The lowest adherence rate is displayed for young adults. Based on a Swedish study [18], we applied an age primary non-adherence rate of 2% for the age group 0-19, 4% for 20-49, 3% for 50-69, and 2% for patients above 70.

### Drug therapies

In the model, we also adjusted for differences in the non-adherence rate between different drug therapies [23-25]. "Short-term" drugs were categorised as the ATC-groups in which the average DDD/patient during the year 2006 was below 200 DDD. In the model, we applied an assumed secondary non-adherence rate of 30% for "Short-term" drugs.

Continuing drug therapies were categorised as ATC-groups in which the average DDD/patient during a year was over 200 DDD. We applied an assumed secondary non-adherence rate of 50% for continuing drug therapies [4-6]. As certain drugs do not have established DDD, we established a third category: ATC-groups with no established DDD. We applied an assumed secondary non-adherence rate of 30% for ATC-groups with no established DDD.

### Multiple medication

In the model, we also adjusted for differences in the secondary non-adherence rate between different numbers of dispensed drugs per patient. Multiple medication is commonly associated with increasing non-adherence [26-32] and, therefore, we assumed that the increase rate in dispensed DDD per patient (primary adherence) decreases with an increased number of prescribed drugs, as well as with an increased rate in ingested DDD per patient (secondary adherence). Due to the lack of information on the actual amount of prescribed drugs, the relation between prescribed and dispensed drugs, with an increasing number of drugs, is unknown. Primary non-adherence rates of 3-50% have been reported [8,12,46]. In our model, we applied the following assumptions of the increasing rate of secondary non-adherence with an increasing number of drugs: 1 to 4 dispensed drugs (DP1-4) per patient has a non-adherence rate of 30%, DP5-9 40%, DP10-14 50%, and DP ≥ 15 60%.

In the result section, we excluded outliers: 7, 262 patients with more than 30 different dispensed drugs during 2006, corresponding to 0.12% of patients with dispensed drugs in Sweden in 2006.

Microsoft Excel (version 5.1.26) was applied for analysis of data. The Regional Ethical Review Board in Linköping, Sweden, approved the study. The average exchange rate 2006 between Euro (€) and Svensk krona (SEK): 1 € = 9.25830 SEK.

## Results

### Total acquisition cost of dispensed drugs

The total acquisition cost of dispensed drugs in Sweden was SEK 23.1 billion in 2006, women, SEK 12.4 and men, SEK 10.7 billion. The average acquisition cost of dispensed drugs per patient was SEK 3, 749, women, SEK 3, 575 and men, SEK 3, 974 (Table 1). Patients' total co-payment was SEK 5.8 billion or 25.2% of total acquisition cost of dispensed drugs 2006.

**Table 1:** Number of individuals, acquisition cost, and acquisition cost per individual in Sweden 2006.

		Individuals			Acq cost (* million SEK)			Acq cost/individual	
	
Age group	All	Women	Men	All	Women	Men	All	Women	Men
0-9	556, 818	263, 385	293, 433	530	194	336	952	736	1, 146
10-19	585, 481	341, 063	244, 418	1, 103	473	630	1, 884	1, 388	2, 577
20-29	650, 272	418, 256	232, 016	1, 162	667	494	1, 786	1, 596	2, 129
30-39	772, 667	456, 855	315, 812	2, 021	1, 186	834	2, 615	2, 596	2, 642
40-49	797, 753	446, 757	350, 996	2, 755	1, 514	1, 241	3, 453	3, 390	3, 534
50-59	905, 910	489, 752	416, 158	3, 983	2, 134	1, 849	4, 397	4, 358	4, 442
60-69	831, 432	436, 085	395, 347	4, 566	2, 354	2, 212	5, 492	5, 399	5, 594
70-79	598, 583	332, 601	265, 982	3, 966	2, 092	1, 874	6, 626	6, 290	7, 046
80-89	392, 433	244, 720	147, 713	2, 638	1, 561	1, 077	6, 721	6, 378	7, 289
≥ 90	70, 324	51, 897	18, 427	375	269	105	5, 326	5, 190	5, 709
Total	6, 161, 673	3, 481, 371	2, 680, 302	23, 098	12, 446	10, 651	3, 749	3, 575	3, 974

Number of individuals, acquisition cost (Acq cost), and acquisition cost per individual for all, women, and men, for different age groups in Sweden 2006.

### Drug acquisition costs with regard to age

Patients < 60 years accounted for 50% of total drug acquisition costs, and patients < 70 years accounted for 2/3 of the total drug acquisition costs. The age groups with the largest proportion of the total costs were 69-69, 50-59, and 70-79 years, with 19.8, 17.2, and 17.2%, respectively.

The average drug acquisition cost per patient increased with an increasing age, from SEK 952 (SEK 736 for women and SEK 1, 146 for men) in the age group 0-9, to a peak in the age group 80-89, with SEK 6, 721 (6, 378 for women and 7, 289 for men). In the age group above 90 years, the drug acquisition cost per patient decreased to a level comparable to the age group 60-69 years (Table 1).

### Drug acquisition costs with regard to drug therapy

The 28 ATC-groups, of a total of 86, with a DDD per patient per year above 200, were categorised as a continuing drug therapy. Continuing drug therapies corresponded to 74.8% of all dispensed DDD and 59.6% of the cost of all dispensed drugs 2006.

The 43 ATC-groups with a DDD per patient per year below 200 were categorised as "short-term" drug therapy. "Short-term" therapy ATC-groups corresponded to 25.2% of all dispensed DDD and 37.4% of the cost of all dispensed drugs in Sweden 2006.

The remaining 15 ATC-groups have no established DDD and corresponded to 3.0% of the total acquisition cost of dispensed drugs.

The 7 ATC-groups with prevalence in the total population above 10% were to be found in both continuing and short-term drug therapies. The ATC-groups with an acquisition cost above SEK one billion were, with one exception, all categorised as continuing therapy.

### Drug acquisition costs with regard to multiple medication

The drug acquisition costs for patients with DP ≥ 5 represented 78.8% of the total drug acquisition costs, patients with DP ≥ 10 and DP ≥ 15, 46.3, and 23.2%, respectively.

**Figure 1:**
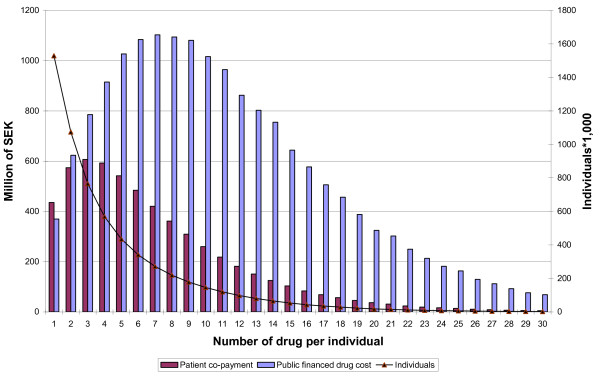
**Number of patients, public financed drug cost and patient co-payment in Sweden 2006**. Number of patients, total amount of public financed drug cost and patient co-payment for patient with 1 to 30 different dispensed drugs in Sweden 2006.

For patients with DP ≥ 5, DP ≥ 10, and DP ≥ 15, the publically financed drug costs represented 80.2, 86.1, and 89.7%, respectively (Figure 1).

The average acquisition cost per patient displayed a near linear relationship with the numbers of dispensed drugs per patient, from an average of SEK 527 for patients with one drug, to SEK 37, 221 for patients with 30 different drugs, women, SEK 429 to SEK 35, 276, and men, SEK 634 to SEK 41, 910.

### Estimates of the drug acquisition costs related to non-adherence

With an assumed primary non-adherence rate for Sweden of 3%, and a secondary non-adherence rate of 50%, the drug acquisition cost related to non-adherence totals SEK 11, 219 million, or 48.5% of total drug acquisition costs in Sweden 2006 (Table 2).

**Table 2:** Drug acquisition costs related to non-adherence with varying therapies and ages in Sweden in 2006.

Age -Therapy	DDD		Acq. Cost		Rate of	Cost of	Rate of	Acq. Cost related to NA	
					
	*million	%	million SEK	%	PNA	prescribe drugs	SNA	million SEK	% of Acq cost
All	5253	100	23154	100	3	23870	50	11219	48, 5
									
Therapy:									
Continuing	3927	74, 8	13798	59, 6	3	14225	50	6686	48, 5
short-term	1326	25, 2	8659	37, 4	3	8927	30	2410	27, 8
(no DDD)			697	3, 0	3	718	30	194	27, 8
Total	5253	100	23154	100, 0		23870		9290	40, 1
									
									
Age:									
0-19	241	4, 6	1641	7, 1	2	1675	50	804	49, 0
20-49	976	18, 6	5966	25, 8	4	6215	50	2859	47, 9
50-69	1894	36, 1	8568	37, 0	3	8833	50	4151	48, 5
70-	2142	40, 8	6979	30, 1	2	7122	50	3418	49, 0
Total	5253	100	23154	100, 0		239844	50	11232	48, 5
									
Therapy and age									
Continuing									
0-19	179		651	2, 8	2	664	50	319	49, 0
20-49	696		3542	15, 3	4	3689	50	1697	47, 9
50-69	1407		5267	22, 7	3	5430	50	2552	48, 5
70-	1645		4339	18, 7	2	4428	50	2125	49, 0
Short-term									
0-19	63		937	4, 0	2	956	30	268	28, 6
20-49	279		2233	9, 6	4	2326	30	605	27, 1
50-69	487		2989	12, 9	3	3081	30	832	27, 8
70-	497		2501	10, 8	2	2552	30	715	28, 6
no DDD									
0-19			53	0, 2	2	54	30	15	28, 6
20-49			192	0, 8	4	200	30	52	27, 1
50-69			313	1, 3	3	322	30	87	27, 8
70-			139	0, 6	2	142	30	40	28, 6
Total	5253	0	23154	100, 0		23844		9306	40, 2

DDD, acquisition costs for dispensed drug (Acq cost), rate of primary non-adherence (PNA), estimated acquisition costs for prescribed drugs, rate of secondary non-adherence (SNA) and drug acquisition costs related to non-adherence (NA) for individuals with varying therapies and ages in Sweden in 2006.

With the assumption of varying secondary non-adherence rates for different types of drug therapies applied in the model, the acquisition cost related to non-adherence amounted to SEK 9, 290 million, or 40.1% of total drug acquisition costs (Table 2).

With the assumption of varying primary non-adherence rates for the different age groups applied in the model, the acquisition cost related to non-adherence amounted to SEK 11, 200 million, or 48.4% of total drug acquisition costs (Table 2).

With the assumption of both different primary non-adherence rates for different age groups, and different secondary non-adherence rates for varying types of drug therapies applied in the model, the acquisition costs related to non-adherence totalled SEK 9, 268 million, or 40.0% of total drug acquisition costs (Table 2).

Finally, when assumptions of different non-adherence rates for an increasing number of drugs were applied in the model, the acquisition costs related to non-adherence totalled SEK 9, 855 million, or 42.6% of total drug acquisition costs (Table 3).

**Table 3:** Drug acquisition costs related to non-adherence with different number of dispensed drugs in Sweden 2006.

			DDD		Acq cost		PNA	Prescribed	SNA	Cost related	non-adherence
							
Therapy	Age	DP	million	%	million	%	rate	drug cost	rate	million	%
Conti.	0-19	DP1-4	106	2	329	1, 4	3	339	40	126	38, 1
		DP5-9	56	1, 1	224	1	2	229	50	110	49, 0
		DP10-14	13	0, 2	61	0, 3	2	63	50	30	49, 0
		DP15-	4	0, 1	36	0, 2	2	37	60	21	59, 2
	20-49	DP1-4	273	5, 2	1151	5	4	1199	40	432	37, 5
		DP5-9	236	4, 5	1301	5, 6	4	1356	50	624	47, 9
		DP10-14	106	2	610	2, 6	4	636	50	292	47, 9
		DP15-	81	1, 5	479	2, 1	4	499	60	279	58, 3
	50-69	DP1-4	250	4, 8	855	3, 7	3	882	40	326	38, 1
		DP5-9	532	10, 1	1820	7, 9	3	1876	50	882	48, 5
		DP10-14	338	6, 4	1286	5, 6	3	1326	50	623	48, 5
		DP15-	288	5, 5	1305	5, 6	3	1346	60	767	58, 8
	70-	DP1-4	118	2, 3	328	1, 4	2	335	40	127	38, 8
		DP5-9	516	9, 8	1276	5, 5	2	1302	50	625	49, 0
		DP10-14	525	10	1337	5, 8	2	1364	50	655	49, 0
		DP15-	485	9, 2	1398	6	2	1426	60	827	59, 2
Short-	0-19	DP1-4	36	0, 7	550	2, 3	2	560	20	101	18, 3
term		DP5-9	20	0, 4	263	1, 1	2	268	30	75	28, 5
		DP10-14	5	0, 1	87	0, 4	2	88	40	33	38, 4
		DP15-	2	0	38	0, 2	2	39	50	19	49, 3
	20-49	DP1-4	97	1, 8	796	3, 4	4	830	20	133	16, 7
		DP5-9	98	1, 8	764	3, 3	4	795	30	207	27, 1
		DP10-14	46	0, 9	363	1, 5	4	379	40	136	37, 6
		DP15-	39	0, 7	310	1, 3	4	323	50	149	47, 9
	50-69	DP1-4	94	1, 8	562	2, 5	3	579	20	98	17, 5
		DP5-9	169	3, 2	1000	4, 3	3	1031	30	278	27, 8
		DP10-14	113	2, 2	692	2, 9	3	714	40	264	38, 2
		DP15-	111	2, 2	734	3, 2	3	756	50	355	48, 4
	70-	DP1-4	39	0, 7	191	0, 8	2	196	20	35	18, 5
		DP5-9	142	2, 8	679	2, 9	2	693	30	194	28, 6
		DP10-14	155	3	763	3, 3	2	778	40	296	38, 7
		DP15-	161	3	868	3, 8	2	886	50	425	49, 0
No DDD			0	0	697	3		718	50	309	44, 3
All			5254	100	23153	99, 9		23848		9855	42, 6

DDD, acquisition costs for dispensed drugs (Acq cost), rate of primary non-adherence (PNA), estimated acquisition costs for prescribed drugs, rate of secondary non-adherence (SNA)and drug acquisition costs related to non-adherence for individuals with varying therapies, age and different number of dispensed drugs in Sweden in 2006

## Discussion

There exists an "enormous amount of quantitative research" concerning medical adherence, but there is no golden standard for measurement [4] and studies of medical adherence vary widely in terminology, definitions and methods [47,48]. Both primary and secondary non-adherence are problems for health care across all therapeutic areas, but the majority of the adherence research has referred to secondary non-adherence and to chronic therapies [21,42]. In 2003, World Health Organisation published "Adherence to long term therapies: evidence for action", which included specific reviews of non-adherence in therapies for asthma, cancer, depression, diabetes, epilepsy, hypertension, tuberculosis [5]. Subsequently, comparing studies of secondary adherence estimates for hypertension, hyperthyroid, type 2 diabetes, seizure disorders, hypercholesterolemia, osteoporosis, and gout [20] and secondary adherence estimate for prostaglandin analogs, statins, bisphosphonates, oral antidiabetics, angiotensin II receptor blockers and overactive bladder medications have being presented [21]. There are also a large number of specific studies and reviews of adherence for specific medical condition e.g. diabetes, hypertensions and dyslipidaemia [49] osteoporosis [50], anticancer treatment [51], HIV [52] and mental disorder [53], etc. The reported results vary widely depending on the disease, study setting and measurements.

As direct measurement of medication consumption is usually not feasible, refill adherence has been applied as an estimate of adherence in population-based studies [54]. However, when medication adherence is estimated from e.g. pharmacy claims databases, the estimates are substantially inflated as non-adherence and early non-persistent patients are largely not included in the estimations [55]. Various studies have reported primary adherence rates of nearly 50% in psoriasis [46], 31.4% in diabetes [14], 24.3% in hypertension [56], and 19.9% in antidepressants [57]. In addition, pharmacy claims databases are not feasible for estimating the secondary adherence (medication taken as prescribed) of "short-term" or "acute" therapies, e.g. all 11 measurements of refill adherence reported by Hess et al are relevant only to secondary adherence for chronic/continuing therapies [43].

### The basic assumptions of non-adherence

Total drug acquisition costs represent between 10-20% of health care expenditure in developed countries [58]. The Swedish rate of 13% is the approximate average within Europe [18]. Consequently, our results appear to be relevant for comparison with the results from other developed countries.

In Sweden, total drug acquisition costs show no clear age profile. The fact that the age group 0-69 years accounted for nearly 70% of costs indicates that the elderly were not the most interesting age group from an economic perspective. Drug acquisition costs per patient increased with increasing age, and reached a peak in the age group 70-89. Women represented somewhat more than 50% of total drug acquisition costs, but men had a higher average cost per patient than women in all age groups.

Continuing drug therapies represented approximately 60% of total drug acquisition costs and were, therefore, the dominant therapy in terms of costs related to non-adherence. "Short-term" drug therapies represented slightly more than one third of total acquisition costs, and had, therefore, a minor impact on total drug costs related to non-adherence.

Multiple medication is a common cause of non-adherence and the rate of non-adherence is assumed to increase with the number of drugs per patient [27-32]. However, we have not found any studies that have assessed, in detail, the relationship between non-adherence and an increasing number of drugs. Therefore, we made the rough assumption, that patients with a large number of different drugs have an above average non-adherence rate, and patients with few drugs have a below average non-adherence rate. However, patients with five or more drugs account for nearly 80% of total drug acquisition costs and have, consequently, a substantial impact on total acquisition costs related to non-adherence.

#### Causes of primary and secondary non-adherence

That patients do not take their medication as prescribed (secondary non-adherence) might be due to several circumstances, e.g. the patients have experienced an adverse drug reaction, patients do not feel that they need the drugs, or they just forget to ingest the drugs. Secondary adherence to drug therapy also depends on the patient's age, gender, disease burden, the complexity and duration of the drug therapy, as well as on the quality of the interaction between patient and prescriber. Furthermore, secondary adherence depends on whether the prescriber follows-up therapy, if the patient feels safe with the drugs, or has multiple providers or multiple medication. Even if differences in the rate of secondary adherence might exist between countries, the causes of secondary adherence are assumed to be universal. Many characteristics of secondary adherence may also have the subsequent effect that patients do not fill their prescriptions (primary non-adherence).

#### Costs related to non-adherence

A factor which, theoretically, is exclusive to primary adherence, is cost-related non-adherence (CRNA), and in many countries CRNA is one of the largest determinants of non-adherence. Sweden and The Netherlands display a high rate of primary adherence compared to most other countries [18,36]. Probably, these countries' low CRNA rates contribute to explaining the high rates of primary adherence. In the US, the average CRNA is 20% or more [17], but for elderly and uninsured adults, the rate is estimated to be 30% and 40%, respectively [7,9,17].

The low rate of CRNA in Sweden (1%) can probably be explained by the comprehensive drug reimbursement system. In excess of a patient co-payment ceiling of SEK 1, 800 (€195), all additional fill of prescriptions are without charge. It could be expected that above this cost ceiling, the patient have no economic incentive to refrain from having the prescriptions filled. Due to the perceived insistence of the health care sector, the patient might feel that filling the prescription is the easiest thing to do, even if the patient, for a number of reasons, does not intend to take the medication.

#### The relationship between primary and secondary non-adherence

Both primary and secondary non-adherence have, independent of each other, an impact on the estimates of drug acquisition costs related to non-adherence, but we can also assume that primary non-adherence and secondary non-adherence impact each other. Without a comprehensive drug reimbursement system, a high rate of secondary non-adherence probably also impacts the rate of primary non-adherence.

A low rate of primary non-adherence combined with a high rate of secondary non-adherence leads to large amounts of unnecessary drug costs, and a high rate of primary non-adherence, combined with a low rate of secondary non-adherence leads to small amounts of, or no, unnecessary drug costs. Consequently, in order to minimize wasteful medical spending, the difference between primary non-adherence and secondary non-adherence should be as limited as possible.

In a public health perspective, the largest cost for the society is untreated diseases and not unnecessary drug sales [59]. Interventions to increase the rate of primary non-adherence to achieve smaller differences compared with the rate of secondary adherence can, therefore, comprise a sub optimising measure, if such intervention also negatively impacts the rate of secondary non-adherence.

### The validity of the estimate of total drug acquisition costs due to non-adherence

In our estimates, non-adherence is determined by varying assumptions regarding the secondary non-adherence rate for age, therapy, and multiple medication. The main determinant is the non-adherence rate of different therapies. However, the proportion of costs referring to non-adherence is approximately 40% of total drug acquisition costs, largely irrespective of any change in the basic assumptions in terms of the non-adherence rate of different therapies. An assumed higher secondary non-adherence rate for "short-term" drug therapies (from 30% to 40%) results in a minor increase in total costs related to non-adherence (from 40 to 44%). If, at the same time, the threshold value for continuing drug therapy is raised (from 200 to 300 DDD per patient per year), total costs related to non-adherence, instead, increase marginally (from 40 to 41%).

The most common non-adherence is for chronic under-use, i.e. patients use less medication than prescribed, or prematurely stop therapy [5], and therefore, we have assumed that the volume of drugs within the non-adherence has no effect on health outcomes. However, it could be assumed that certain amounts of the ingested drugs, but which are not taken in full according to the prescription or recommendation might have some positive effect on the health outcome. This assumption will not change the estimate of drug acquisition costs related to non-adherence, but can be seen as an argument that the definition of non-adherence to drug therapies might overestimate the volume of unnecessary drug sales.

### The relevance of total drug acquisition costs due to non-adherence

That patients' non-adherence to therapy leads to high extra costs for society in terms of poor health for patients [37], and to the fact that as approximately 40% of the total drug acquisition costs is related to patients' non-adherence to drug therapies, this must be seen to comprise a highly non-satisfactory situation. As the amount of total drug acquisition costs represented approximately a third of the total cost of primary care in Sweden in 2006 [60], this should also be considered by the health care stakeholders as being highly relevant in an economic perspective.

### Implications for health care

Non-adherence to drug therapies represents a waste of large amounts of economic resources, both for the patients and for society. A reduction of drug acquisition costs related to non-adherence should be achieved by interventions focused on improving secondary adherence, e.g. active handling to change patient behaviour, reduce side effects and therapy complexity, improving the provision of health services, and improving the interaction between health care personnel and patients.

Interventions should be directed to patients or to providers, or health care managers. Sadly, long term patient-focused intervention has shown to have no substantial effect on adherence [5], e.g. attempts with different types of patient reminding-systems for filling of prescriptions have shown no significant effects [61-63], but the routine follow-up by the prescriber has displayed significant increasing adherence [11,64]. An intervention directed to prescribers to decrease the complexity of the drug therapy, e.g. a change to one dosage a day instead of two, has also improved adherence [5]. Another intervention that have been suggested is the introduction of financial incentives or other rewards for patients who adhere to therapy [65].

A low rate of primary non-adherence not combined with a low rate of secondary non-adherence results in major costs for patients and society in terms of unnecessary drug sales. A reduction of the cost related to non-adherence can, therefore, also be achieved by interventions aimed at increasing primary non-adherence, e.g. modifying the drug reimbursement system to better correspond with patients' secondary non-adherence.

### Strengths and limitations

The study is based on patient data on pharmacy claims without any sample, recall or interview bias for all patients with dispensed drugs in an entire national population. The study combined actual pharmacy claims data with general assumptions based on previous studies of different non-adherence rates for patients in different age groups, drug therapies, and with a varying number of dispensed drugs. A limitation is that the assumptions made in the present study are based on previous studies that often only focus on adherence to one specific long-term medical therapy. Results from previous studies concerning the non-adherence rate for short-term therapies, patients' age, and number of dispensed drugs per patient are often only qualitative. Consequently, certain of the assumptions about varying non-adherence rates in the present study are relatively rough. Furthermore, the register includes all dispensed prescriptions drugs but has no information regarding other types of drugs e.g. OTC-drugs and CAM. Nor has the register any information about the volume and the distribution of prescribed drugs that are not filled, nor as regards the rate of filled drug not taken as prescribed

## Conclusions

Our estimate indicates that drug acquisition costs related to non-adherence represent a substantial proportion of the economic resources in the health care sector. A low rate of primary non-adherence, combined with a high rate of secondary non-adherence, contributes to a large amount of unnecessary medical spending. Thus, efforts of different types of interventions are needed to improve secondary adherence.

## Competing interests

The authors declare that they have no competing interests.

## Authors' contributions

Both authors participated in the design of the study and the discussion of the findings. BH executed the data management and BH drafted the manuscript. GP revised the manuscript. Both authors read and approved the final manuscript.

## Pre-publication history

The pre-publication history for this paper can be accessed here:

http://www.biomedcentral.com/1472-6963/11/326/prepub
